# Comparative genome analysis of *Lactobacillus casei* strains isolated from Actimel and Yakult products reveals marked similarities and points to a common origin

**DOI:** 10.1111/1751-7915.12062

**Published:** 2013-07-01

**Authors:** François P Douillard, Ravi Kant, Jarmo Ritari, Lars Paulin, Airi Palva, Willem M Vos

**Affiliations:** 1 Department of Veterinary Biosciences, University of HelsinkiHelsinki, Finland; 2 Institute of Biotechnology, University of HelsinkiHelsinki, Finland; 3 Infection Biology Program, Department of Bacteriology and Immunology, Haartman Institute, University of HelsinkiHelsinki, Finland; 4 Laboratory of Microbiology, Wageningen UniversityWageningen, the Netherlands

## Abstract

The members of the *Lactobacillus* genus are widely used in the food and feed industry and show a remarkable ecological adaptability. Several *Lactobacillus* strains have been marketed as probiotics as they possess health-promoting properties for the host. In the present study, we used two complementary next-generation sequencing technologies to deduce the genome sequences of two *Lactobacillus casei* strains LcA and LcY, which were isolated from the products Actimel and Yakult, commercialized as probiotics. The LcA and LcY draft genomes have, respectively, an estimated size of 3067 and 3082 Mb and a G+C content of 46.3%. Both strains are close to identical to each other and differ by no more than minor chromosomal re-arrangements, substitutions, insertions and deletions, as evident from the verified presence of one insertion-deletion (InDel) and only 29 single-nucleotide polymorphisms (SNPs). In terms of coding capacity, LcA and LcY are predicted to encode a comparable exoproteome, indicating that LcA and LcY are likely to establish similar interactions with human intestinal cells. Moreover, both *L. casei* LcA and LcY harboured a 59.6 kb plasmid that shared high similarities with plasmids found in other *L. casei* strains, such as W56 and BD-II. Further analysis revealed that the *L. casei* plasmids constitute a good evolution marker within the *L. casei* species. The plasmids of the LcA and LcY strains are almost identical, as testified by the presence of only three verified SNPs, and share a 3.5 kb region encoding a remnant of a lactose PTS system that is absent from the plasmids of W56 and BD-II but conserved in another smaller *L. casei* plasmid (pLC2W). Our observations imply that the results obtained in animal and human experiments performed with the Actimel and Yakult strains can be compared with each other as these strains share a very recent common ancestor.

**Funding Information** The present work was supported by the Center of Excellence in Microbial Food Safety Research (Academy of Finland, Grant 141140), Grant ERC 250172 – Microbes Inside from the European Research Council and Grants 137389 and 141130 from the Academy of Finland. F.P.D. was funded by a postdoctoral research fellowship (Academy of Finland, Grant 252123).

## Introduction

Lactobacilli belong to the lactic acid bacteria (LAB) group and constitute a very large genus of Gram-positive, non-sporulating bacteria that show remarkable ecological adaptability and phylogenetic diversity (Axelsson, 2004). Commonly found in human body cavities, i.e. vagina, intestinal tract and oral cavity, they also naturally persist in a broad range of food environments, such as fermented milk, meat and plant (Kandler and Weiss, 1986). A number of lactobacilli species have been extensively implemented in various industrial processes, as starter or adjunct cultures (Stiles and Holzapfel, 1997). More recently, some strains of the species *Lactobacillus casei*, *L. paracasei*, *L. reuteri*, *L. rhamnosus*, *L. acidophilus, L. plantarum* and *L. johnsonii* have also been increasingly employed in food products marketed as probiotics (de Vos, 2011). The health benefits associated with the consumption of products containing some of these commercialized *Lactobacillus* strains have been demonstrated by a number of human clinical studies on patients with diverse disorders (Kalliomaki *et al*., 2003; Saxelin *et al*., 2005; Sykora *et al*., 2005; Almeida *et al*., 2012; Hajela *et al*., 2012). However, the large diversity of lactobacilli also suggested that probiotic functions and health-promoting properties are specific to each strain, justifying the need to examine probiotic lactobacilli strains individually on a genomic and molecular basis (Ventura *et al*., 2009). In this regard, the *L. casei* group is of particular interest, as it constitutes an important reservoir of probiotic-marketed strains. Although subject to recurrent and controversial changes over its taxonomy and nomenclature, the *L. casei* group currently consists of three species: *L. casei*, *L. paracasei* and *L. rhamnosus* (Collins *et al*., 1989; Klein *et al*., 1998; Dellaglio *et al*., 2002; Dobson *et al*., 2004; Sato *et al*., 2012). The *L. casei* group includes some well-documented strains, such as *L. rhamnosus* strain GG (Kankainen *et al*., 2009; Lebeer *et al*., 2012) or *L. casei* strain BL23 (Maze *et al*., 2010). Although not known to be commercialized, *L. casei* strain BL23 possesses potential probiotic functions which relate to its anti-inflammatory properties in an animal model (Foligne *et al*., 2007; Rochat *et al*., 2007) and its ability to bind extracellular matrix *in vitro* (Munoz-Provencio *et al*., 2010). A majority of *L. casei* strains are also resistant to stresses, such as exposure to acid and bile salts present in the gastrointestinal tract, a trait characteristic of probiotic strains (Broadbent *et al*., 2010; Alcántara and Zúñiga, 2012; Hamon *et al*., 2012).

Peculiarly, the genomes of some probiotic *L. casei* strains widely used in probiotic products have not been examined. Up to date, only six complete *L. casei* genomes have been sequenced: *L. casei* ATCC 334 (Makarova *et al*., 2006), *L. casei* BL23 (Maze *et al*., 2010), *L. casei* Zhang (Zhang *et al*., 2007), *L. casei* LCW2 (Chen *et al*., 2011), *L. casei* W56 (Hochwind *et al*., 2012) and *L. casei* BD-II (Ai *et al*., 2011) and 12 additional *L. casei* genomes have been partially sequenced (NCBI database, as on 1 March 2013). Recent comparative genomic analyses of the *L. casei* species provided important and valuable insights into the species diversity in regards to its ecological versatility and genome evolution (Cai *et al*., 2009; Broadbent *et al*., 2012). Broadbent and colleagues also demonstrated that the presence of other bacterial species residing in *L. casei* ecological habitats clearly impact the *L. casei* pan-genome, indicating that the Distributed Genome Hypothesis can be applied to non-pathogenic bacterial species, such as *L. casei*. This also illustrated the significant role of horizontal gene transfer events in lifestyle adaptation (Broadbent *et al*., 2012). Moreover, the significant genome decay observed in some *L. casei* strains adapted to the dairy environment, is believed to contribute to the diversity of the species (Broadbent *et al*., 2012).

Recently, we addressed the genome stability of *Lactobacillus* strains, used in industrial processes (Douillard *et al*., 2013). By genomic re-sequencing of product isolates of strain *L. rhamnosus* GG, we determined the stability of this widely used probiotic strain (Douillard *et al*., 2013). Identical probiotic functions were detected in strains isolated from products from *bona fide* producers, testifying for the stability of *L. rhamnosus* GG, which has a well-characterized 3.01 Mb genome (Kankainen *et al*., 2009). However, a different situation may be encountered when these or other strains are not handled appropriately by the manufacturer. This seems to be the case for some unspecified products with *L. rhamnosus* strains, as reported recently, although post-sampling events cannot be excluded (Sybesma *et al*., 2013). Specifically, a deletion was identified in a region encoding the genes for the production of extracellular pili that bind to human intestinal mucus (Kankainen *et al*., 2009; Reunanen *et al*., 2012; Sybesma *et al*., 2013). In contrast, we showed that *L. casei* strains isolated from two globally marketed products, Actimel and Yakult, contained pili genes that were highly conserved in sequence to that of *L. rhamnosus* GG but did not express these pili under the tested conditions (Douillard *et al*., 2013). This was explained by the absence of an IS-mediated promoter that drives constitutive pili expression in *L. rhamnosus* GG (Douillard *et al*., 2013). The present study expands on this by providing a further detailed comparative genomic characterization of these two *L. casei* strains that derive from strains branded as *L. casei defensis* and *L. casei* Shirota. It shows that these strains are highly similar though having a reportedly different history. Among others this is illustrated by the presence of 29 confirmed SNPs and 1 verified InDel, an identical predicted exoproteome and a ∼59 kb plasmid that is nearly identical in both strains.

## Results and discussion

### Genomic features of *L. casei* strain LcY isolated from the Yakult product

**Figure 1:**
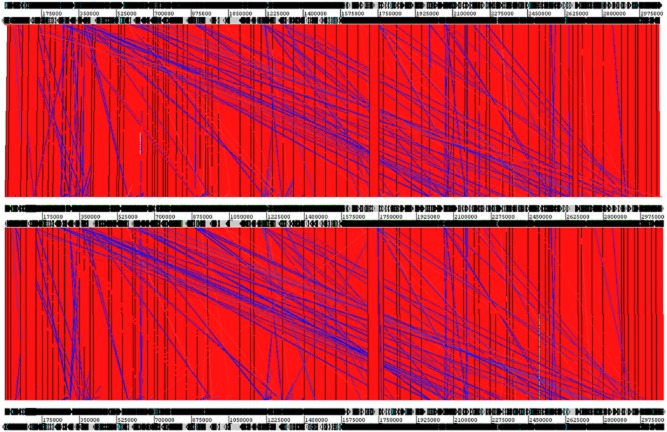
Comparison of the genomes of *L. casei* LcA, LcY and BL23 using ACT (Artemis Comparison Tool). LcY, BL23 and LcA are respectively shown from top to bottom in the figure. Bars designate conserved chromosomal regions between LcA, LcY and BL23 (BlastN hits) that have the same orientation (red) or have been inverted (blue).

**Figure 2:**
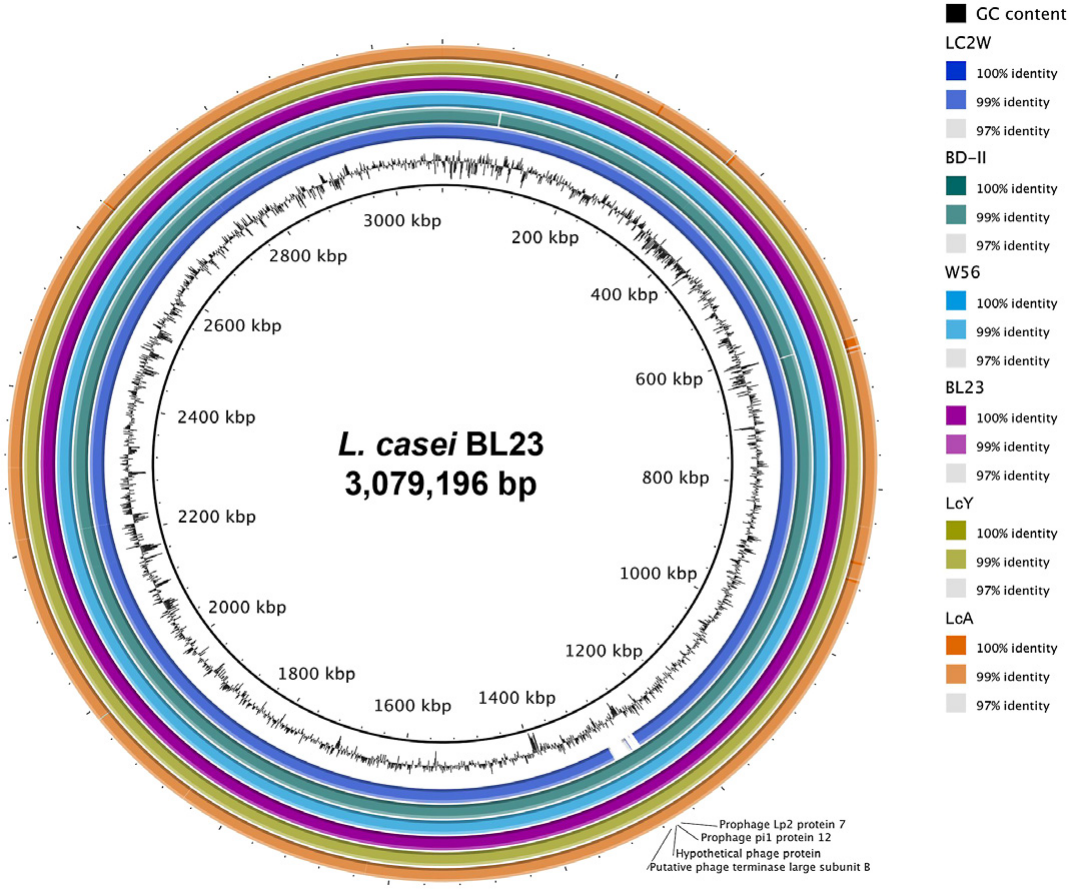
BLAST Ring Image Generator (BRIG) analysis of *L. casei* strains LcA, LcY, BD-II, W56, LC2W and BL23. *Lactobacillus casei* strain BL23 was used as the reference genome. Colours indicate the percentage of sequence identity.

The probiotic-marketed product Yakult is commercialized by Yakult Honsha (Japan) and contains *L. casei* strain Shirota that has previously been subject to multiple studies with regards to its potential health-promoting properties in humans and animals (Rochat *et al*., 2007; Matsumoto *et al*., 2010; Naito *et al*., 2011; Nanno *et al*., 2011; Almeida *et al*., 2012). In the present study, we sequenced the genome of *L. casei* strain LcY that had been isolated from the Yakult product (Douillard *et al*., 2013) and is assumed to represent *L. casei* strain Shirota. Genomic DNA from *L. casei* LcY was isolated and sequenced by a combination of two next-generation sequencing platforms 454 GS-FLX+ (Life Sciences) and SOLiD (Life Technologies). The contig order and orientation were determined using *L. casei* BL23, W56, LC2W and BD-II as reference genomes (Maze *et al*., 2010; Ai *et al*., 2011; Chen *et al*., 2011; Hochwind *et al*., 2012), allowing us to assemble the *de novo* contigs into one single scaffold (Table 1). The thus constructed *L. casei* LcY draft genome consists of one circular chromosome with an estimated size of 3 082 048 bp and an overall GC content of 46.33%. It also harbours an estimated 59.6 kb plasmid designated pYAK that shows high sequence identity with plasmids pW56 and pBD-II respectively present in *L. casei* strains W56 and BD-II (Ai *et al*., 2011; Hochwind *et al*., 2012). Initial automated annotation of the *L. casei* LcY genome (and LcA; see below) was performed using RAST (Aziz *et al*., 2008). However, due to the very high sequence identity and synteny between some *L. casei* strains (Figs 1 and 2), we re-annotated both genomes using the Rapid Annotation Transfer Tool (RATT) (Aziz *et al*., 2008), as we observed that this tool provided more accurate gene annotations (data not shown). A total of 3119 coding DNA sequences (CDS) were predicted and annotated using RATT (Otto *et al*., 2011), including 3044 proteins (Table 1). Five rRNA operons, containing 5S, 16S and 23S rRNA genes, are scattered throughout the genome of LcY. The first three rRNA operons are located in the leading strand and the other two on the lagging strand. Forty-nine tRNAs were located in the vicinity of the rRNA genes, whereas the other 11 tRNAs were uniformly distributed on the chromosome. Interestingly, three unique tRNAs species were identified, e.g. tRNAs for cysteine, histidine and tryptophane.

**Table 1:** General genomic features of *L. casei* strains LcA and LcY isolated from Actimel and Yakult probiotic-marketed products

Genetic features	*L. casei* LcY	*L. casei* LcA	*L. casei* BL23
Origin	Yakult	Actimel	N/A
Number of scaffolds	1	1	1
Estimated chromosome size	3 082 048	3 066 955	3 079 196
GC content (%)	46.33	46.34	46.34
Coding DNA sequences (CDS)	3 119	3 109	3 119
Protein coding sequences	3 044	3 031	3 044
RNA genes	75	78	75
rRNA genes	15	16	15
tRNA genes	60	62	60
Plasmids	1	1	0
CRISPR locus	1	1	1

Data shown in the table have been estimated based on draft genome assembly and RATT transfer gene annotation. Features relative to the plasmids pACT and pYAK respectively present in strains LcA and LcY have not been incorporated in the table.

N/A, not available.

### Genomic features of *L. casei* strain LcA isolated from the Actimel product

The probiotic-marketed drinkable yogurt Actimel is produced by Danone and contains *L. casei* strain DN-114001, branded as *L. casei defensis*. *Lactobacillus casei* strain DN-114001 has been studied for its potential probiotic properties in various animal and human studies (Marcos *et al*., 2004; Sykora *et al*., 2005; Pawlowska *et al*., 2007; Guillemard *et al*., 2010). We previously isolated and characterized the *L. casei* strain LcA present in the Actimel product (Douillard *et al*., 2013). We assume that *L. casei* LcA is virtually identical to the seed culture of *L. casei* strain DN-114001. Using two next-generation sequencing platforms, we determined the genome sequence of *L. casei* strain LcA similarly as described above (Table 1). The thus obtained draft genome of *L. casei* LcA consists of one scaffold with an estimated size of 3 066 955 bp and a 59.6 kb plasmid (pACT). The overall G+C content of LcA chromosome is 46.34%. A total of 3109 CDS were predicted and annotated using RATT (Otto *et al*., 2011), including 3031 proteins (Table 1). The total number of CDS for both strains LcA and LcY is comparable to BL23 (3119 CDS) and in line with previous CDS range prediction in lactobacilli (2700–3700) (Makarova *et al*., 2006). Five rRNA operons, containing 5S, 16S and 23S rRNA genes, and 62 tRNA genes are present the genome of *L. casei* LcA and show a similar distribution as in *L. casei* LcY. Three tRNAs species were unique in LcA, e.g. tRNAs for cysteine, histidine and tryptophane and tyrosine. To reduce the analysis load, we did not address the exact number of pseudogenes and IS elements in this study.

### Genetic relatedness and comparison with other members of the *L. casei* species

**Figure 3:**
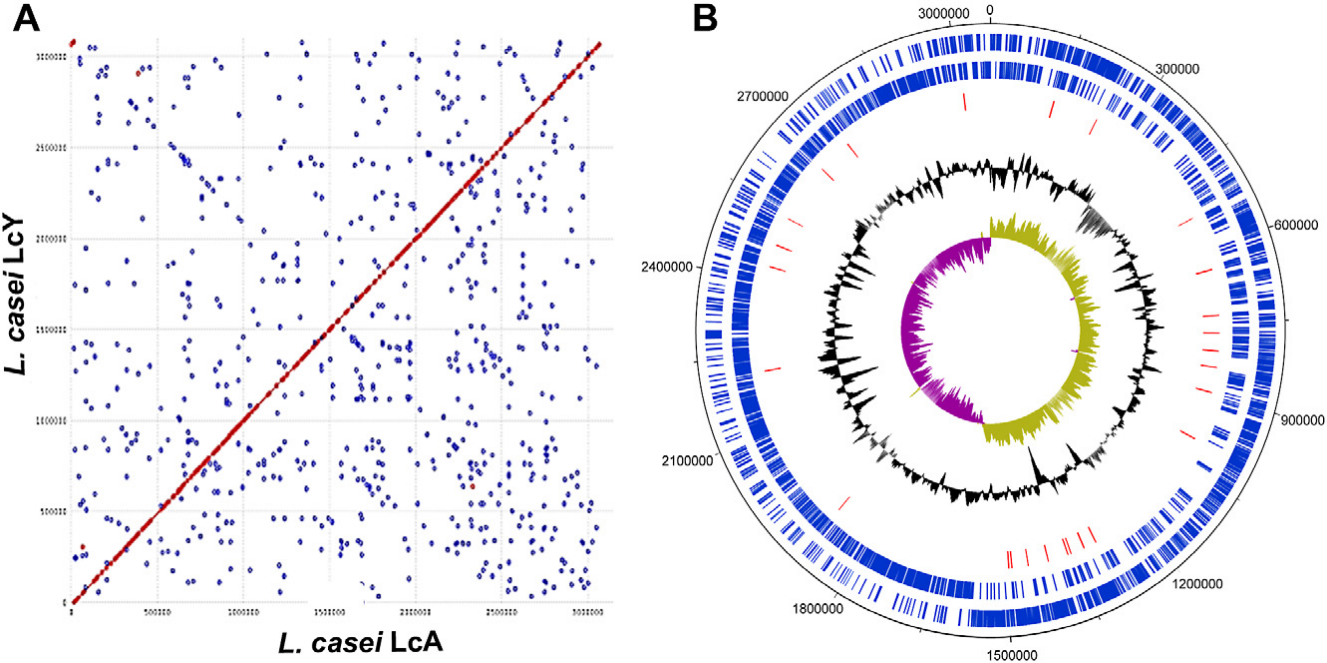
Comparison of the genomes of *L. casei* strains LcA and LcY.A. Synteny plot shows the comparison at the nucleotide level of the genome of *L. casei* LcY (vertical axis) with the genome of *L. casei* LcA (horizontal axis). Red dots indicate homologous chromosomal regions between genomes with the same orientation. Blue dots indicate homologous chromosomal regions which are in the opposite direction.B. Genome circle of *L. casei* LcA using DNAPlotter (Carver *et al*., 2009). Blue bars indicate CDS and red bars indicate SNPs and InDels identified when compared with *L. casei* LcY.

In terms of genomic features, there were clear similarities between the two strains, i.e. genome size, GC% content and CDS number (Table 1). We first compared *L. casei* LcA and LcY to *L. casei* BL23 as the latter strain also has been found to have potential probiotic properties, although it is not commercialized. Genomic alignments of *L. casei* strains LcA, LcY and BL23 further showed a high degree of synteny with large identical chromosomal blocks, indicating that genome differences are mostly due to minor genetic recombination events (Fig. 1). Further comparative genomic analysis of the genomes of *L. casei* LcA, LcY and other *L. casei* strains revealed their close genetic relatedness (Fig. 2), which is in line with a previous comparative genomic study on the *L. casei* species (Broadbent *et al*., 2012). In the species *L. casei*, six distinct sublineages were defined and one of them consists of three closely related strains BL23, BD-II and LC2W (Broadbent *et al*., 2012). Our data indicate that *L. casei* strains LcA, LcY and the recently sequenced strain W56 also belong to that very same cluster, whereas other sequenced *L. casei* strains, such as strain Zhang and ATCC334 distinctly evolved from the common ancestor. *Lactobacillus casei* strains LcA and LcY are highly similar to each other, as shown by synteny plot (Fig. 3A). However, some small differences were observed between *L. casei* LcA and LcY genomes. When comparing the number of shared genes between *L. casei* LcA and LcY, it is estimated that 3031 genes were present in both strains, which represent 99.6% of the gene content of strain BL23 (Maze *et al*., 2010). All genes of strain BL23 were found in *L. casei* LcY, whereas 13 genes present in strain BL23 were missing in LcA based on RATT analysis. These 13 genes that were predicted to encode putative proteins and transposases are likely missed due the present state of the genomes, since repetitive elements is one of the most common cause for assembly gaps (Kingsford *et al*., 2010). These results also suggest that *L. casei* LcA, LcY and BL23 have a virtually similar coding capacity. We then used both SOLiD and 454 data to look at the presence of any SNPs and InDels between the two *L. casei* draft genomes. SOLiD reads of LcA were mapped to the sequence of LcY and vice versa. Identical SNPs and InDels identified in both mappings were then manually inspected. We were able then to confidently identify the presence of one InDel and a total of 26 SNPs (Table 2 and Fig. 3B). The InDel found in LcA strain is located in the gene LCACT_2629 (*gltB*), encoding a NADPH-dependent glutamate synthase and it results in the truncation of the protein. Four of the SNPs are located in intercistronic regions, while the remaining ones did not generate any stop codons and only gave rise to some missense mutations, testifying for the similarity of the strains (Table 2). This is confirmed by the high degree of metabolic similarity that we described previously and indicates that the small differences in efficiency of sugar utilization are likely to be explained by experimental variations (Douillard *et al*., 2013).

**Table 2:** List of unequivocal SNPS and InDels identified and verified between LcA and LcY genomes, chromosome and plasmid included

LcY coordinate	Base	Base	LcA coordinate	AA change LcY/LcA	Locus tag and predicted gene product
**Chromosome**					
137132	T	G	136805	N14H	LCACT_0132/LCY_0132, putative protein
236020	A	T	235693	N340I	LCACT_0235/LCY_0235, inulosucrase (fragment)
530929	G	A	527365	Q82Q	LCACT_0522/LCY_0523, ABC transporter
649781	C	A	641841	N/A	Intercistronic region
649813	T	G	641373	N/A	Intercistronic region
744458	A	C	736004	Y135D	LCACT_0725/LCY_0737, tagatose 1,6-diphosphate aldolase
780579	T	C	772125	I39M	LCACT_0759/LCY_0771, putative protein
817971	G	T	809517	R140R	LCACT_0800/LCY_0812, HAD-superfamily hydrolase
852271	A	G	843663	E13E	LCACT_0832/LCY_0844, thioredoxin
904463	A	G	895886	G26G	LCACT_0880/LCY_0892, putative protein
1010123	G	A	996844	R136K	LCACT_0988/LCY_1000, ABC transporter
1314821	A	G	1300766	K76K	LCACT_1311/LCY_1323, beta-lactamase family protein
1342691	A	T	1328636	I5F	LCACT_1341/LCY_1353, cysteine desulfurase
1372643	T	G	1358501	N/A	Intercistronic region
1379614	A	G	1365472	F97L	LCACT_1383/LCY_1395, ABC transporter related
1423524	C	T	1409467	P107L	LCACT_1434/LCY_1446, methyltransferase
1467351	A	G	1453376	T219A	LCACT_1475/LCY_1487, acetyltransferase
1502680	C	G	1488607	N/A	Intercistronic region
1508926	G	A	1494853	D338N	LCACT_1510/LCY_1522, acyltransferase
1901798	C	A	1886618	L313I	LCACT_1895/LCY_1907, sensor protein
2231304	G	A	2217739	G199D	LCACT_2201/LCY_2214, putative protein
2447647	T	C	2433375	N64S	LCACT_2405/LCY_2418, oxidoreductase
2494439	G	T	2480167	P166Q	LCACT_2449/LCY_2462, uridine phosphorylase
2554046	G	T	2539774	G76V	LCACT_2511/LCY_2524, transcriptional regulator
2754290	T	G	2740097	Q51P	LCACT_2701/LCY_2714, glutathione reductase
3025404	C	T	3011097	R383Q	LCACT_2974/LCY_2987, malate oxidoreductase
2678962	A	.	2664876	Frameshift	LCACT_2629/LCY_2642, NADPH-dependent glutamate synthase (truncation in LCACT_2629)
**Plasmid**					
168	C	A	168	N/A	Intercistronic region
27281	T	C	27281	Y691Y	pACT_0028/pYAK_0028, hypothetical protein
37993	G	A	37993	N/A	Intercistronic region

The SNPs and InDels were identified based on reciproqual SOLiD mapping to each draft genome sequence of *L. casei* strains LcA and LcY.

N/A, not available.

### Plasmids pACT and pYAK

**Figure 4:**
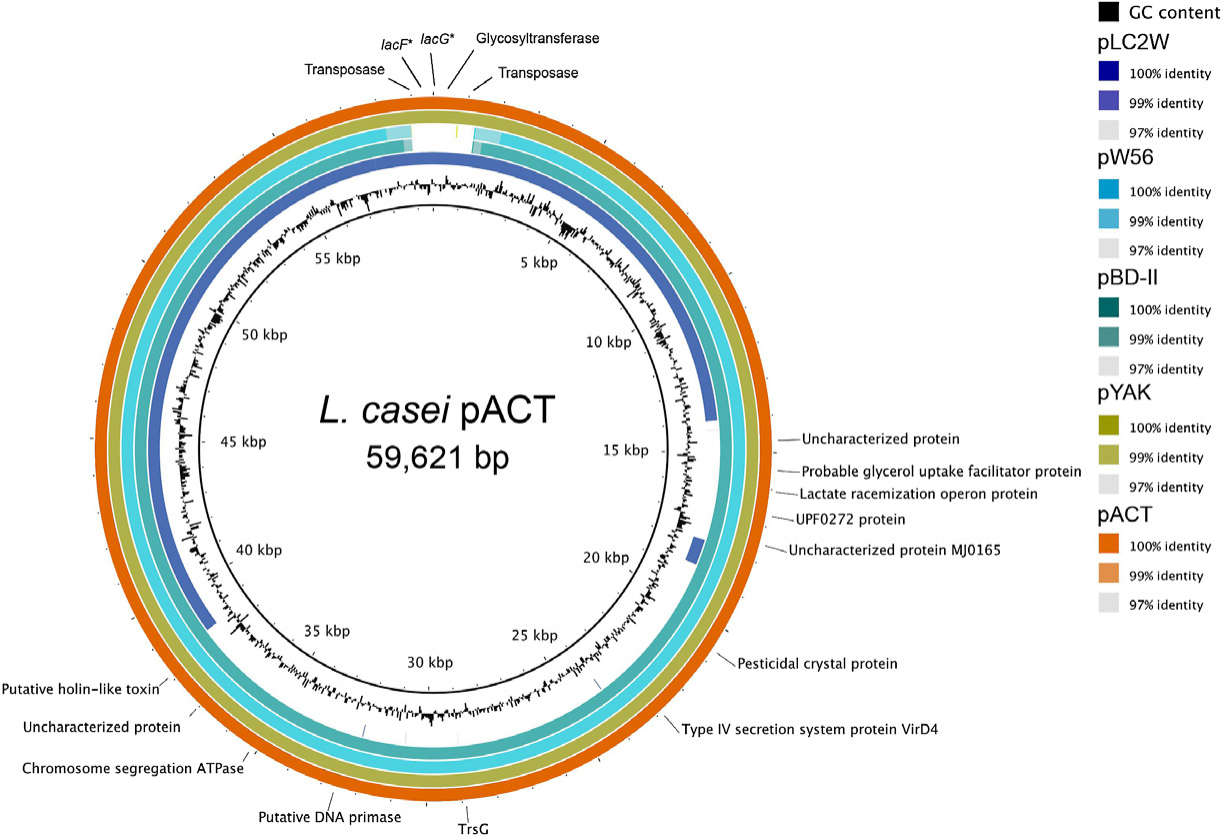
BLAST Ring Image Generator (BRIG) analysis of the plasmids present in *L. casei* strains LcA, LcY, W56, BD-II and Lc2W. Plasmid of the *L. casei* strain LcA was used as the plasmid backbone. Colours indicate the percentage of sequence identity. Genes present in the variable regions are also indicated on the figure. Genes marked with an asterisk are altered.

Genome sequencing of both *L. casei* strains LcA and LcY also revealed the presence of one 59.6 kb plasmid, respectively, named pACT and pYAK. PCR amplification was used to confirm the presence of the plasmid in LcA and LcY by joining all the contigs into a single circular molecule. Our data suggest that plasmids pACT and pYAK are nearly identical in terms of size and gene content and highly similar to two previously reported plasmids found in *L. casei* strains W56 and BD-II (Ai *et al*., 2011; Hochwind *et al*., 2012). The plasmid draft sequences were carefully compared using a similar approach as mentioned above. Three SNPs were detected and confirmed (Table 2). pACT and pYAK share all sequences present in the 38 kb plasmid pLC2W found in *L. casei* strain LC2W (Ai *et al*., 2011), indicating that a major deletion occurred in this plasmid (Fig. 4). Remarkably, this shared 38 kb region includes a 3.5 kb fragment not present in plasmids pW56 and pBD-II (Fig. 4). This fragment contains a remnant of lactose PTS system. No significant homologies between pACT, pYAK and plasmids from *L. casei* strains Zhang and ATCC334 were found. In spite of the recent sequencing of strains W56, BD-II and LC2W, their respective plasmids remained poorly annotated and comprehended. Similarly, plasmids pYAK and pACT are also predicted to encode mostly hypothetical proteins, transposases and a restriction–modification enzyme system. We did not observed any unique functional traits that are observed in LAB and in Lactobacilli, such a lactose hydrolysis (Cai *et al*., 2009) or copper resistance (Barré *et al*., 2007). To further explore the properties of these plasmids, comparative functional analysis of these strains and their plasmid-cured derivatives would possibly reveal some features of interest. It is noteworthy that a higher diversity was observed within the *L. casei* plasmids pACT, pYAK, pW56, pBD-II and pLC2W than within the chromosomes of the same *L. casei* strains, suggesting that plasmids evolves faster than the genomes and are suitable markers to differentiate *L. casei* strains (Fig. 4). Similar observations have recently been reported for *Burkholderia* and *Vibrio* (Cooper *et al*., 2010).

### Environmental traits

Chromosomal sequences that are known to be highly variable can be found in the CRISPR (clustered regularly interspaced short palindromic repeats) loci that are found in almost half of the prokaryotic genomes (Horvath and Barrangou, 2010). The CRISPRs and their associated *cas*-genes from a prokaryotic immune system and play an important role in regulating horizontal gene transfers. It has been well documented that some bacteria acquired a CRISPR locus as it provides protection and immunization against plasmids, phages and other mobile elements (Horvath and Barrangou, 2010; Marraffini and Sontheimer, 2010; Makarova *et al*., 2011). We identified the presence of a Type II-A/LsaI1 CRISPR*-cas* system in the genome of both *L. casei* LcA and LcY, consisting of four *cas* genes and an array of 21 spacers interspaced by a 36 bp direct repeat. Identical CRISPR locus could also be found in BL23, BD-II and LC2W (Ai *et al*., 2011; Hochwind *et al*., 2012; Broadbent *et al*., 2012). Another relevant property for industrial strains marketed as probiotics is their ability to endure a number of stresses that are typically encountered during production and also in the gastrointestinal tract. In the genomes of both *L. casei* LcA and LcY, we identified genes encoding heat (i.e. GroEL and GroES), cold (i.e. CspA and CspG) and alkaline shock proteins, SOS-response proteins and Clp proteases (ClpE, ClpX, ClpL and ClpP).

### Prediction of the exoproteome, cell-surface proteins and other relevant functions

Proteins expressed on the bacterial cell surface play essential roles in bacteria–host cross-talk, virulence, colonization, immunogenicity and have been proposed to constitute probiotic traits. Using the SignalP prediction model, a total of 162 proteins were predicted to have a peptide signal in both *L. casei* LcA and LcY, indicating that they may be translocated across the cellular membrane and constitute the exoproteome. LcA and LcY strains possess a comparable set of secreted proteins. A total of 47% of these were predicted to be cell wall associated but have not been annotated, underlining the fact that a number of interaction players remain to be uncovered. Recent studies performed in *L. casei* identified new candidate proteins that could contribute to the bacteria–host interaction. For example, a gene encoding a fibronectin/fibrinogen-binding protein FbpA in BL23 was found to be involved in the attachment of the bacterial cells with the intestinal epithelium (Munoz-Provencio *et al*., 2010). Interestingly, an FbpA homologue was also encoded in both LcA and LcY genomes (LCACT_1599 and LCY_1611 respectively). Two other proteins of interest found in LcA and LcY are p40 (LCACT_0271; LCY_0271) and p75 (LCACT_0023; LCY_0023), and are identical to the two major secreted proteins in *L. rhamnosus* GG (Claes *et al*., 2012). These proteins have attracted considerable attention as they were found to display anti-apoptotic and cellular protective properties on the intestinal epithelium in *L. rhamnosus* GG (Yan *et al*., 2007) and *L. casei* BL23 (Bauerl *et al*., 2010). Other predicted secreted proteins (23%) were mostly associated with ABC transporters and phosphotransferase systems essential in the cell metabolism.

We further examined the genes encoding LPxTG or LPxTG-like proteins, as they typically have a pivotal role in virulence, colonization or persistence in ecological niches. Cell-wall-associated LPxTG proteins typically possess a C-terminal sorting signal domain consisting of a highly conserved LPxTG motif flanked by a chain of hydrophobic and positively charged amino acidic residues (Navarre and Schneewind, 1999; Mazmanian *et al*., 2001). The LPxTG motif is specifically recognized by membrane-associated sortases that cleave the LPXTG motif between the threonine (T) residue and the glycine (G) residue and then covalently anchors the proteins to the peptidoglycan layer (Schneewind and Missiakas, 2012). Although LPxTG proteins are well documented in Gram-positive pathogens, their roles in commensal intestinal lactobacilli only begin to be comprehended. In strains LcA and LcY, these include enzymes, adhesive glycoproteins and pilin-specific proteins (Table 3). Notably, six of them were encoding pili subunits and were clustered in two distinct pili operons, *spaFED* and *spaCBA*, termed after their homologous genes in *L. rhamnosus* GG (Kankainen *et al*., 2009). Both operons showed a similar genetic order where the three pilin-specific genes were associated with a pilin-specific sortase. Similarly, the strain LcA has these two pili gene clusters (Table 3). The genetic organization of the pili operon is widespread within the *L. casei* species, as previously noted (Broadbent *et al*., 2012). Despite the presence of pili operons, a recent study demonstrated that the strains LcA, LcY and BL23 did not express mucus-binding pili *in vitro* (Douillard *et al*., 2013). In *L. rhamnosus* GG, the orthologous pili gene cluster *spaCBA-srtC* is expressed and confers strong mucus-binding abilities to the host (Kankainen *et al*., 2009; Lebeer *et al*., 2012; Reunanen *et al*., 2012). We showed that the insertion of an IS element upstream the *spaC* adhesion pilin gene constituted a functional promoter in *L. rhamnosus* GG, whereas in the *L. casei* strains it is absent, explaining the lack of pili production under conditions that resemble industrial and intestinal conditions (Douillard *et al*., 2013).

**Table 3:** List of predicted proteins in *L. casei* strains LcA and LcY harbouring a LPxTG or LPxTG-like motif

Gene name	Predicted gene product	Gene name
*Strain LcA*		*Strain LcY*
LCACT_0280	β-*N*-acetylglucosaminidase precursor	LCY_0280
LCACT_0513	Pilus-specific adhesion protein (SpaC)	LCY_0514
LCACT_0514	Pilus-specific minor-backbone protein (SpaB)	LCY_0515
LCACT_0515	Pilus-specific major-backbone protein (SpaA)	LCY_0516
LCACT_0525	Cell envelope-associated proteinase	LCY_0526
LCACT_0526	Cell envelope-associated proteinase (PrtR)	LCY_0527
LCACT_0651	Hypothetical protein	LCY_0663
LCACT_1204	Hypothetical protein	LCY_1216
LCACT_2049	Hypothetical protein	LCY_2061
LCACT_2373	Cell-wall-associated serine proteinase	LCY_2386
LCACT_2425	Mucus-binding factor (MBF)	LCY_2438
LCACT_2463	Pilus-specific adhesion protein (SpaF)	LCY_2476
LCACT_2462	Pilus-specific minor backbone protein (SpaE)	LCY_2475
LCACT_2461	Pilus-specific major-backbone protein (SpaD)	LCY_2474
LCACT_2524	Outer membrane protein	LCY_2537

The proteins were identified using SignalP (Petersen *et al*., 2011) and CW-PRED (Litou *et al*., 2008) prediction methods.

Reports on the function of other LPxTG proteins in lactobacilli are still sparse, in spite of the fact that several species contain strains that are marketed as probiotics, i.e. *L. plantarum*, *L. rhamnosus*, *L. salivarius*. Notably, two housekeeping sortases genes were identified in *L. casei* LcA and LcY, similar as in strain BL23 (Munoz-Provencio *et al*., 2012). In the latter strain, it was found that the housekeeping sortases SrtA are required for the cell wall anchoring of *N*-acetylglucosaminidases and proteinases (Munoz-Provencio *et al*., 2012). In addition to the pilin-specific proteins mentioned above, *L. casei* LcA and LcY are predicted to encode nine LPxTG and LPxTG-like proteins that are covalently coupled to the bacterial cell wall. It is noteworthy that the LPxTG motif is highly conserved in all these proteins along with the presence of hydrophobic tail essential for their retention on the cell surface prior to their sortase-mediated cell wall anchoring (Perry *et al*., 2002; Hendrickx *et al*., 2011). Most of these have no known function, but it is reasonable to think that these surface-anchored proteins may be involved in the intestinal colonization and lifestyle of *L. casei*. Interestingly, the genomes of both *L. casei* LcA and LcY also are predicted to encode the Mucus-Binding Factor (MBF). The MBF protein is well conserved in *L. casei* strains but also in the *L. rhamnosus* strains, where it has an orthologue that was shown to be involved in adhesion mechanisms in the intestinal tract (von Ossowski *et al*., 2011). However, the MBF does not seem to play a significant role in *L. casei* LcA and LcY, as both strains did not show any mucus-binding ability *in vitro* (Douillard *et al*., 2013).

### Conclusions

*Lactobacillus casei* strains LcA and LcY are genomically closely related to a number of other *L. casei* strains, including the well-studied *L. casei* BL23. Comparative analysis of the *L. casei* plasmids revealed a greater diversity than found at the chromosomal level, indicating that, in spite of their extrachromosomal nature, plasmids are a good indicator of the evolution of *L. casei* species and further substantiates the high identity between LcA, LcY and other *L. casei* strains. *Lactobacillus casei* LcA and LcY contain a comparable set of genes encoding secreted proteins, underlining that they may display similar probiotic functions to the host. The limited number of SNPs and InDels detected in both chromosome and plasmid (in total 29 SNPs and one InDel) indicates that the strains LcA and LcY are very closely related. We also cannot exclude the possibility that some of this limited amount of diversity was generated during the strain isolation procedure. The genomic sequences reported in the present study offer a functional basis to facilitate the understanding of the well-known *L. casei* strains LcA and LcY. Our observations also imply that the results obtained in animal and human experimental studies performed with the *L. casei* strains used in Actimel and Yakult products may be compared with each other as these two strains share a very recent common ancestor.

## Experimental procedures

### Bacterial strains, growth conditions and DNA isolation

The two *L. casei* strains LcA and LcY were isolated from the products Yakult (Yakult Honsha, Japan) and Actimel (Danone, France), as previously described (Douillard *et al*., 2013). Bacterial strains were cultured in MRS broth at 37°C in anaerobic conditions overnight and 2 ml of cell suspension was used for genomic DNA isolation using Wizard Genomic DNA Purification Kit (Promega, WI, USA) as per the manufacturer's instructions.

### Genome sequencing, assembly, annotation and analysis

The genomes of *L. casei* strains LcA and LcY were sequenced using two next-generation sequencing platforms (454 GS-FLX+, Life Sciences and SOLiD, Life Technologies) and assembled *de novo* at the Institute of Biotechnology (Helsinki, Finland). The combination of both platforms allowed a much greater sequencing depth and accuracy in contrast to a unique sequencing technology approach, as previously done for *L. casei* strains LcA and LcY (Douillard *et al*., 2013). The 454 coverage on basis of numbers of reads in contigs is averaging 14X for both LcA and LcY genomes and the SOLiD coverage estimated from mappings is of ∼88X for LcA and ∼107X for LcY. Using progressive Mauve genome alignment package (Darling *et al*., 2010), the draft contigs were ordered and oriented. The published genomes of *L. casei* BL23, W56, BD-II and LC2W were used as references, improving the draft genome assemblies. Automatic genome annotation was conducted using RAST pipelines (Aziz *et al*., 2008) and subsequently RATT (Otto *et al*., 2011). PCR amplification from total DNA was performed to confirm contig order and circularity of the plasmids identified in both *L. casei* strains LcA and LcY. Pseudogenes and transposases were not addressed in the present study. Signal sequences were predicted with SignalP v4.0 (Petersen *et al*., 2011) and LPxTG proteins were identified using CW-PRED (Litou *et al*., 2008). BLAST Ring maps were generated using BRIG (Alikhan *et al*., 2011). SNPs and InDels analysis of both draft genomes was performed as follows. The LcA SOLiD reads were mapped to the LcY draft genome sequence using SHRiMP2, and BAM files of the mapping result were generated using SAMtools (Li *et al*., 2009; Rumble *et al*., 2009). Similarly, the LcY SOLiD reads were mapped to the LcA draft genome sequence. SNPs and InDels were detected with the MUMmer software package using nucmer parameters ‘maxmatch’ and ‘-c 100’, and show-snps command was used with parameter ‘-C’ (Kurtz *et al*., 2004). Only unequivocal SNPs and InDels detected in both mappings were reported. Read mappings and annotations were inspected with Artemis (Carver *et al*., 2012).

### Sequence data deposition

The Whole Genome Shotgun projects for *L. casei* strains LcA and LcY have been deposited at DDBJ/EMBL/GenBank under the accession AQPP00000000 and ARNV00000000, respectively. The versions of LcA and LcY draft genomes described in this paper are the first versions, AQPP01000000 and ARNV01000000, respectively. *L. casei* strains LcA and LcY are available from Prof. W. M. de Vos, Department of Veterinary Biosciences, University of Helsinki, Helsinki, Finland. Draft genome and plasmid annotations presented in this study are accessible in the Supporting Information.

## Supporting information

File S1 Draft genome annotation of LcA chromosome.

File S2 Draft genome annotation of pACT plasmid.

File S3 Draft genome annotation of LcY chromosome.

File S4 Draft genome annotation of pYAK plasmid.
