# Corrigendum

**DOI:** 10.1111/1751-7915.12095

**Published:** 2013-12-10

**Comparative genome analysis of *Lactobacillus casei* strains isolated from Actimel and Yakult products reveals marked similarities and points to a common origin**

François P. Douillard, Ravi Kant, Jarmo Ritari, Lars Paulin, Airi Palva and Willem M. de Vos

In the draft genome sequence of *Lactobacillus casei* strain LcY originally published by Douillard and colleagues (2013), it was reported that this strain contained a ∼ 40 kb prophage region. This is incorrect and strain LcY is devoid of this prophage as based on re-analysis of both SOLiD and 454 sequencing data as well as polymerase chain reaction analysis. The genomes of *L. casei* strains LcA and LcY are highly similar to each other and also to some other *L. casei* isolates. Hence, the conclusions of our manuscript are unchanged except for the absence of a prophage in LcY. Their comparable predicted exoproteome also suggests that *L. casei* LcA and LcY may possess similar probiotic properties, legitimating the comparison of the *in vivo* studies performed with the *L.* *casei* strains used in Actimel and Yakult products. The LcY Whole Genome Shotgun project has been deposited at DDBJ/EMBL/GenBank under the accession ARNV00000000. The revised version described relative to this paper is version ARNV02000000.
